# Recycling and Environmental Sustainability in Anesthesia Practice: Beyond Low‑Flow Anesthesia

**DOI:** 10.7759/cureus.101037

**Published:** 2026-01-07

**Authors:** Saad Nadeem, Muhammad Zain, Yusuf Islam, Sharif Mohamed

**Affiliations:** 1 Internal Medicine, Texas A&M College of Medicine, Houston, USA; 2 Internal Medicine, Texas A&M College of Medicine, Bryan, USA; 3 Imaging Physics, University of Texas MD Anderson Cancer Center, Houston, USA; 4 Anesthesiology, University of Texas Medical Branch, Galveston, USA

**Keywords:** anesthesia waste, cost savings, healthcare sustainability, operating room recycling, public and environmental health, regulated medical waste, sustainability outcomes

## Abstract

Operating rooms generate substantial amounts of solid waste and greenhouse gas emissions from anesthetic agents, yet most “green anesthesia” efforts have focused narrowly on low‑flow volatile delivery rather than on the broader waste stream associated with anesthetic care. A large proportion of anesthesia‑adjacent materials (e.g., blue wrap, intravenous (IV) containers, and packaging) are clean, potentially recyclable items that are frequently misclassified as regulated medical waste, inflating environmental and cost-related impact and disposal costs.​

A narrative review of the literature was conducted using PubMed as the primary database, supplemented by manual citation tracking. Search terms combined concepts such as “operating room waste,” “anesthesia waste,” “blue wrap,” “recycling,” “volatile anesthetics,” and “low‑flow anesthesia.” Studies were included if they reported on the composition of operating room or anesthesia‑related waste, the capacity for recycling perioperative materials, the implementation of recycling or waste‑segregation programs, cost outcomes associated with regulated medical waste, or emission-reduction tactics for inhaled anesthetic emissions; commentaries without primary data were excluded. This approach yielded several dozen relevant observational audits, quality‑improvement reports, implementation case studies, surveys, and life‑cycle analyses published over the past two decades.​

Across waste audits, anesthesiology accounts for roughly one‑quarter of operating room waste, with approximately half to two‑thirds of anesthesia‑related solid waste classified as clean and potentially recyclable plastics and paper products. High‑yield streams include polypropylene (PP) blue wrap, IV fluid containers (high-density polyethylene (HDPE)/PP/polyvinyl chloride (PVC)), rigid plastic basins, flexible plastic packaging, and paper/cardboard, for which targeted recycling programs have achieved substantial diversion with minimal workflow disruption. Multi‑site and single‑center recycling campaigns report large reductions in regulated medical waste volumes, cost avoidance from lower disposal fees, and, in some cases, direct revenue from the sale of recyclables. In parallel, life‑cycle studies of volatile anesthetics demonstrate that desflurane and nitrous oxide contribute disproportionately to the anesthetic carbon footprint, whereas low‑flow delivery, preferential selection of lower‑GWP (global warming potential) agents, reduced nitrous oxide use, and gas capture technologies markedly lower emissions without impairing patient safety.

An integrated strategy that couples point‑of‑use recycling of uncontaminated anesthesia‑adjacent materials with standardized low‑flow anesthetic protocols and gas‑mitigation technologies presents a feasible, high‑yield pathway to improve environmental performance and control costs in perioperative care. Departments have to prioritize mapping local recyclability, enhancing waste handling segregation and signage, embedding recycling receptacles into anesthesia workflows, and tracking both diversion and emissions metrics to guide iterative quality improvement.​

## Introduction and background

Operating rooms (ORs) are among the most resource‑intensive areas in hospitals, generating disproportionately high volumes of waste and greenhouse gas emissions relative to their physical footprint. Published life‑cycle and waste‑audit studies report that perioperative services can contribute roughly 20-30% of a hospital’s total waste, and anesthetic care is responsible for approximately 20-25% of OR waste, a large proportion of which is plastic and packaging that is technically recyclable but frequently discarded as general or regulated medical waste (RMW). Volatile anesthetic agents further add to this footprint; desflurane, for example, has a 100‑year global warming potential on the order of 2,500 times that of carbon dioxide, and nitrous oxide has an atmospheric lifetime measured in decades, so these gases represent a measurable share of healthcare‑associated greenhouse gas emissions despite being used in relatively small volumes [1,2].

To date, much of the “green anesthesia” discourse has focused on inhaled anesthetic gases, particularly the adoption of low‑flow techniques and the preferential use of lower‑GWP (global warming potential) agents, as primary strategies to reduce the carbon footprint of anesthesia practice. However, a substantial and underused opportunity lies in the proper segregation, disposal, and recycling of non‑contaminated OR materials, such as outer packaging films, paperboard inserts, blue wrap, and rigid intravenous (IV) containers, which contribute considerable solid‑waste volumes in large institutions and increase disposal costs when misclassified as RMW. Multiple audits and provider surveys show that a large fraction of anesthesia‑adjacent materials are clean and technically recyclable, yet they are routinely discarded into RMW streams because of infection‑control concerns, ambiguous signage, and staff knowledge gaps, leading to demonstrable environmental and financial inefficiencies rather than merely perceived ones [1,2].

Existing reviews and guidelines frequently cover either gas‑related emissions or general surgical waste, but few synthesize anesthesia‑specific recycling opportunities alongside gas‑mitigation strategies in a single, pragmatic framework for perioperative teams. The specific gap this review intends to address is the lack of an integrated, anesthesia‑focused overview that compares the environmental and cost consequences of OR waste streams and volatile anesthetic agents and highlights practical, scalable interventions beyond low‑flow anesthesia alone. Therefore, the objective of this explanatory review is to describe the composition and the potential for recycling of anesthesia‑related OR waste, summarize the implementation and cost outcomes of recycling and waste‑segregation programs, and synthesize current evidence on volatile anesthetic reduction approaches, with the goal of outlining a combined pathway to improve green sustainability in anesthesia practice [1,3].

## Review

Methods

This article is a narrative review that aims to synthesize current knowledge on OR waste, recycling opportunities, and the environmental impacts of anesthesia‑related solid waste and volatile anesthetic gases. The review focuses on anesthesia‑adjacent materials and practices, including OR waste composition, the potential for recycling of common materials, implementation of recycling and waste‑segregation programs, cost outcomes associated with RMW, and reduction approaches for inhaled anesthetic agents.​

A structured literature search was executed using PubMed as the primary database. The search included articles published from January 2000 through October 2025 to capture contemporary perioperative practice and sustainability initiatives. Search terms combined Medical Subject Headings (MeSH) and keywords such as “operating room waste,” “perioperative waste,” “anesthesia waste,” “blue wrap,” “plastic waste,” “recycling,” “regulated medical waste,” “green sustainability,” “volatile anesthetics,” “low‑flow anesthesia,” and “life cycle assessment.” The reference lists of key articles and relevant reviews were manually screened to identify additional studies and implementation reports that met the aims of this review.​

Studies were included if they addressed at least one of the following domains: anesthesia‑related waste generation or composition; recyclability or segregation of perioperative materials (e.g., blue wrap, IV containers, rigid basins, flexible packaging); implementation of OR recycling or waste‑segregation programs; economic impacts of waste management (such as RMW volumes, disposal costs, cost avoidance, or revenue from recyclables); or environmental impacts and reduction approaches related to volatile anesthetics, low‑flow techniques, or gas‑capture technologies. Commentaries and opinion pieces without primary data, and articles focused solely on non‑perioperative hospital waste, were excluded to preserve pertinence to anesthesia practice.​

The included literature encompassed observational waste audits, cross‑sectional surveys, quality‑improvement and implementation reports, life‑cycle assessments, and selected narrative or scoping reviews that contributed empirical or synthesized data to the above domains. Given the heterogeneity of study designs and outcomes, a formal tool‑based risk‑of‑bias assessment was not applied. Instead, studies were appraised qualitatively, with attention to clarity of methods, transparency of baseline and follow‑up measurements, and the plausibility of reported environmental and economic results.

Results

Composition and Volume of OR Waste

On average, ORs generate significant waste, with anesthesiology accounting for nearly 25% of total OR waste [2-7]. The majority of perioperative waste consists of plastics, of which almost 60% of anesthesia-related waste is potentially recyclable and does not come into contact with the patient [5-7]. Predominant materials include blue wrap, rigid plastic containers, flexible packaging, IV fluid containers, and paper-based packaging materials [5,8,9].

Blue Wrap

Among the predominant materials, blue wrap, which is commonly composed of polypropylene (PP) (#5 plastic), accounts for the single most significant recyclable waste stream in the OR, at 20% of total OR waste [5,9]. Blue wrap is ubiquitously used to wrap surgical instrument sets and implant trays, and some national estimates indicate that nearly 255 million pounds of blue wrap are discarded annually in US healthcare facilities [5]. When properly segregated before patient contact or contamination, blue wrap maintains its classification as a clean recyclable material suitable for standard PP recycling streams [5,7]. The uniform composition and high-volume characteristics of the material make it an ideal target for recycling initiatives, as demonstrated by multiple institutional programs [5,9].

IV Fluid Containers

IV fluid containers represent another significant source of recyclable waste [5,9]. Most receptacles are manufactured from high-density polyethylene (HDPE) or PP, both of which are cited as recyclable materials [5,7]. Northwestern Medicine recently conducted a pilot program to recycle IV bags [5]. The program directed coordination across multiple hospital departments, including nursing, supply chain management, and environmental services, to help establish new proposed collection protocols within the existing clinical workflow. The study successfully shifted over 12,000 pounds of polyvinyl chloride (PVC) IV bags during the initial implementation phase, underscoring the noteworthy volume of this single material type [5].

Rigid Plastic Containers

Another standard material used throughout anesthesia care is rigid plastic containers, including pitchers, trays, basins, and graduated cylinders. These materials are commonly manufactured from recyclable plastics (HDPE or PP) [5-7]. Despite their recyclable nature, these materials are frequently discarded as general waste due to a lack of infrastructure for degradation and the OR staff's lack of awareness of alternative disposal pathways [6,7]. Waste audits have noted that 3-8% of total OR waste volume consists of plastic containers, representing a readily accessible recycling opportunity with minimal workflow disruption [7,8].

Flexible Plastic Packaging

Flexible plastic packaging materials, including sterile airway wraps, single-use anesthesia equipment, and breathing circuits, also constitute nearly 10-15% of OR waste [5-9]. Most of these materials are polyethylene, which makes them recyclable to an extent [5-9]. While considered a secondary target compared to blue wrap and rigid container recycling, several advanced recycling programs have successfully incorporated flexible plastic packaging into their collection protocols [5-9].

Anesthetic care generates a diverse stream of waste products, with significant contributions from blue wrap, IV fluid containers, rigid plastic basins and containers, and flexible and non-flexible plastic packaging [5-9]. As detailed in Table 1, blue wraps, among all other materials, constitute approximately 20% of OR waste and are highly recyclable [5-9]. In comparison, IV fluid containers and rigid plastics together account for an additional 8-18%, both of which are readily recyclable when properly segregated [5-9]. Flexible plastic packaging accounts for 10-15% of OR waste and, while recyclable, often presents logistical challenges due to its material composition [5-9]. Collectively, these streams demonstrate that targeted recycling interventions, particularly those focused on high-volume uncontaminated materials, can divert a substantial proportion of waste in an OR setting [5-9].

**Table 1:** Composition and Recyclability of Major Operating Room Waste Streams HDPE, high-density polyethylene; PP, polypropylene; OR, operating room.

Material Type	Plastic Type	% of OR Waste	Recyclability Status
Blue wrap (polypropylene)	Polypropylene	~20%	Highly recyclable [5-9]
IV fluid containers	HDPE, PP	Variable (5-10%)	Highly recyclable [5-9]
Rigid plastic basins/containers	HDPE, PP	Variable (3-8%)	Highly recyclable [5-9]
Flexible plastic packaging	Polyethylene	Variable (10-15%)	Recyclable [5-9]
Syringe packaging	Mixed plastics	Variable (5-8%)	Limited recyclability [5-9]
Paper/cardboard packaging	Paper fiber	~15-20%	Highly recyclable [5-9]

Impact of Educational and Behavioral Interventions

Among anesthesia providers, convenience is a critical determinant of recycling participation, with multiple studies highlighting that proximity and ease of access to recycling receptacles increase recycling rates in the OR. A national Canadian survey of anesthesiologists (n≈250 respondents) found that although 97.5% expressed willingness to recycle, only 30.2% reported actually doing so; 63.5% cited a lack of convenient recycling facilities, and 62.8% cited inadequate information or education as key barriers. In a similar survey of anesthesiologists from Australia, New Zealand, and England, 93% supported increased OR recycling, but only 11% reported recycling within their own ORs; 49% identified inadequate facilities, particularly the lack of accessible receptacles in the OR workspace, as a major barrier. Qualitative work and implementation reports further show that workflow‑oriented changes, such as placing recycling bags or bins directly on anesthesia carts, reduce cognitive load and increase habitual recycling participation, and most providers describe environmental restructuring as essential for adoption of sustainable practices [10-13]. Overall, these findings indicate that although anesthesiologists are highly motivated, practical barriers to workflow integration and convenience remain the primary obstacles to effective OR recycling, and relatively simple environmental and educational interventions can meaningfully improve participation [10-13].​

Waste Audits and Cost Savings

Comprehensive waste audits have established that OR recycling programs can be both environmentally beneficial and economically favorable, with environmental gains often aligning with substantial cost savings for healthcare organizations. The economic rationale is largely driven by the differential disposal costs of various OR waste streams, as RMW typically costs three to five times more to dispose of than general waste because of transportation, incineration, and specialized handling requirements [14]. This cost differential creates a strong financial incentive to divert clean, recyclable materials away from expensive RMW streams.

The Cleveland Clinic neurosurgery department conducted an eight‑week pilot program focused on blue wrap recycling, demonstrating both environmental and financial benefits. Over a 39‑day active collection period, the program diverted more than 1,247 pounds of blue wrap from landfill, averaging 32 pounds per day [14]. The initial investment totaled $14,987 (baling machines $11,200, transport carts $3,697), and the program achieved $31,680 in cost avoidance from reduced waste disposal fees, along with $100 in direct revenue from selling baled blue wrap at approximately $0.08 per pound [14]. When extrapolated across all 20 ORs at the institution, the projected annual cost avoidance totaled $174,240, with approximately $5,000 in revenue from recyclables [14].

In another initiative, Baxter International partnered with Northwestern Medicine to pilot the first hospital‑based IV bag recycling program in the United States. During the initial implementation phase, the program diverted more than 12,000 pounds of PVC IV bags from landfills [15]. Although detailed cost data were not reported, the program demonstrated feasibility, avoided disposal fees, and showed that a previously non‑recycled stream could be recovered and processed into industrial products such as protective edging materials, floor mats, and medical‑grade plastic models [8,15,16].

Broader institutional initiatives have reported similar economic benefits. Inova Fairfax Hospital, an 833‑bed tertiary care center, implemented an RMW reduction protocol across its ORs and achieved an 18.6% reduction in RMW over six months, with associated cost savings exceeding $15,000 [15-17]. Magee‑Womens Hospital of UPMC reduced RMW by 47% in OR and labor‑and‑delivery units, diverting 28,795 pounds of waste in a single year and realizing more than $89,000 in annual savings. Mills‑Peninsula Medical Center in California transitioned from disposable blue wrap to reusable rigid sterilization containers and recovered its $34,987 investment within 8.2 months; first‑year savings totaled $16,186, with additional annual savings of more than $25,000 from eliminated blue wrap purchases [15-17]. The Universitary Hospital Mútua Terrassa in Spain implemented a segregation and recycling program across hospital disciplines, combining education, infrastructure, and feedback; over 19 months, they reported an 85% reduction in waste‑related emissions, with a weekly carbon footprint of 79.1 kg CO₂‑equivalent [15-17]. Cork University Maternity Hospital in Ireland developed a surgical wrap recycling program that collected 282.1 m² of PP wrap over 66 operations during a five‑week pilot, with projections suggesting annual recycling of 11,564 m² and an estimated reduction of 2.2 tons of CO₂ emissions [7,14,15,17].

Taken together, these largely single‑center audits and quality‑improvement projects show that OR recycling programs can reach financial breakeven within one to two years and generate annual savings, while also reducing RMW volumes and emissions. As summarized in Table 2, there is heterogeneity in program design, duration, and scale, but most report meaningful waste diversion and cost avoidance. The financial benefits arise from multiple sources, including reduced RMW disposal costs, occasional revenue from recyclable material sales, decreased purchasing of disposable items when reusable alternatives are adopted, and avoided costs of expanding waste management infrastructure.

**Table 2:** Economic and Environmental Impact of OR Recycling Programs PVC, polyvinyl chloride; RMW, regulated medical waste; IV, intravenous; OR, operating room.

Institution/Study	Duration	Waste Diverted	Cost Savings/Impact
Cleveland Clinic neurosurgery pilot	39 days	1,247 lbs blue wrap	$31,680 cost avoidance [14-17]
Northwestern Medicine IV bag pilot	Phase 1 pilot	12,000 lbs PVC IV bags	Not quantified [14-17]
Inova Fairfax Hospital	6 months	18.6% RMW reduction	>$15,000 [14-17]
Magee-Womens Hospital UPMC	1 year	28,795 lbs RMW	>$89,000 [14-17]
Mills-Peninsula Medical Center	1 year	Blue wrap to rigid containers	$16,186 first year [14-17]
Universitary Hospital Mútua Terrassa	19 months	85% waste-related emissions reduction	79.1 kg CO₂e/week [14-17]
Cork University Maternity Hospital	Pilot phase	Weekly bales of blue wrap	Ongoing collection [14-17]

Volatile Anesthetic Agents: Environmental and Life Cycle Outcomes

The environmental impact of anesthetic agents is highly significant. This is because volatile agents such as sevoflurane, isoflurane, and desflurane significantly impact the atmosphere due to their potent greenhouse gas properties and relatively long atmospheric lifetimes [18-20]. Desflurane, in particular, has a 100-year global warming potential (GWP₁₀₀) of around 2540. These inhalational anesthetics account for about 3% of all healthcare greenhouse gas emissions, with volatile agents ranking among the most significant environmental contributors. Life‑cycle investigations confirm that the chief environmental burden comes from anesthetic gas released during clinical use, with the impacts of transportation and manufacturing contributing minimally.

One of the root issues of volatile agents is that very little is metabolized in vivo. For example, only 0.02% of desflurane is typically metabolized before exhalation. Moreover, the limited use of gas-capture or scavenging technologies results in significant gas release into the atmosphere during the anesthetic process. This escaped desflurane would persist in the atmosphere for 21 years, whereas nitrous oxide, used as an adjunct, can persist for over 100 years; both gases amplify global warming. Many comparative studies indicate that the type of volatile agent, along with the fresh gas flow rate, dictates the environmental damage caused by anesthetic agents [18-23]. Use of low‑flow anesthesia (0.5 L/min or less) has been shown to reduce excess emissions by over 40% without affecting anesthetic depth or patient safety, and educational interventions can markedly reduce desflurane usage. New vapor‑capture systems, such as membrane‑based absorbers, have also shown promising results in reducing gas waste by up to 10-fold. When considered alongside solid‑waste interventions, such as recycling of blue wrap and IV containers and improved segregation of non‑contaminated plastics, these gas‑focused strategies complement waste‑reduction efforts, together forming a combined approach to lowering both the carbon footprint and waste‑related impacts of anesthesia practice [3,18-21,24,25].

Barriers and Facilitators to Sustainability

The incorporation of environmentally sustainable measures in anesthesia faces many complex barriers. These issues include a lack of education on waste management and environmental protocols, cost constraints, and misconceptions. This is compounded by infection control concerns, limited space in the OR, and entrenched procedural habits in clinical settings, which hinder the implementation and adoption of environmentally sustainable practices. Among all OR waste, anesthesiology accounts for around 25%; however, more than half of these materials are potentially recyclable. Moreover, significant unnecessary incineration occurs in the OR due to the misclassification of uncontaminated waste. Inadequate staff education in the OR is a significant obstacle to the adoption and maintenance of recycling stations.

Several recent hospital programs have successfully adopted green, sustainable anesthesia practices, overcoming these barriers. Within these programs, high levels of leadership involvement from department chairs and surgical service lines were a significant factor, allowing the organization to prioritize sustainability [24-27]. The participation of department leaders has also been crucial to overcoming inadequate funding and creating structured education programs [24-27]. Moreover, resident‑led programs and departmental “green rounds” are effective in reducing anesthetic waste and increasing recycling of materials such as blue wrap and IV containers [24-27]. Targeted educational modules have also reduced desflurane use. These findings emphasize that provider capability (knowledge and skills), opportunity (infrastructure, space, and workflow design), and motivation (professional and institutional priorities) all need to be addressed for sustainable practices to be adopted and sustained, which is consistent with simple behavior‑change models [16,17,24-27]. In high‑resource healthcare systems, this may include investment in reusable sterilization systems, comprehensive recycling infrastructure, and gas‑capture technologies, whereas in low‑ and middle‑income settings, more feasible starting points often include low‑flow protocols, basic segregation of non‑contaminated plastic and paper waste, and low‑cost educational interventions tailored to local constraints [3,16,17,24-27].

Sustainability Outcomes and Future Directions

The adoption of environmentally sustainable measures in the OR has been shown to yield measurable environmental and financial benefits. An intervention adopting low‑flow protocols has demonstrated reductions of approximately 50% in anesthetic gas usage and 42% in equivalent CO₂ emissions [27]. Targeted OR‑specific recycling programs focusing on OR plastics and PP blue wraps have also reduced thousands of kilograms of non‑contaminated waste from RMW streams, with associated cost avoidance and lower disposal‑related emissions [27]. Other programs incorporating volatile gas‑capture systems have demonstrated the ability to recover up to 70% of excess exhaled anesthetic vapors, additionally reducing the anesthetic carbon footprint [19,28]. Collectively, these interventions suggest that combining low‑flow anesthesia, gas selection and capture, and solid‑waste recycling can produce substantial sustainability gains, although most reports are short‑ to medium‑term quality‑improvement projects and longer‑term durability remains less certain [19,28].

The key to improving environmental efficiency measures and outcomes in the OR is enforcing standardized policies, adapting them to the local context, and thoughtfully applying innovative technologies. Worldwide guidelines, such as the European Union’s 2026 F‑gas regulation and the UK National Health Service’s desflurane phase‑out, demonstrate a rising regulatory pledge to eco-friendly operations; early reports indicate reductions in high‑GWP anesthetic use and a shift toward lower‑impact agents in participating systems, although detailed, long‑term outcome data are still emerging and may not be directly generalizable to other regions [29]. Moreover, new technologies, such as life‑cycle assessment tools integrated into perioperative electronic records, can help present environmental impact data at the point of care, guiding anesthetic choice in real time and supporting transitions toward more sustainable options [29]. However, these strategies commonly require infrastructure, informatics capacity, and capital investment that may be challenging in low‑ and middle‑income settings, where simpler measures, such as basic low‑flow protocols, targeted education, and low‑cost waste‑segregation initiatives, are likely to be more feasible initial steps toward sustained environmental viability [29].

Discussion

Anesthesiology reflects the broader healthcare system and the difficulty of balancing new sustainability measures with patient safety and clinical efficiency. The traditional focus on reducing volatile anesthetic use has expanded attention to the full life cycle of waste produced by anesthetic practice in the OR, underscoring the need for an integrated approach to both gas emissions and solid‑waste management. This review highlights that it is important to look beyond low‑flow anesthesia alone and consider a wider range of green sustainability options, including waste segregation, recycling, and procurement strategies, alongside gas‑focused interventions [18-21].

Emerging evidence indicates substantial financial and ecological impacts of misclassified physical waste in the OR. Multiple reviews and audits demonstrate that physical waste, particularly uncontaminated plastics and blue wrap, constitutes a major environmental and economic burden, even if its direct greenhouse gas impact differs from that of volatile anesthetics. Most notably, almost 60% of anesthesia‑related waste is potentially recyclable and does not come into contact with the patient, yet institutional obstacles within the OR, such as ambiguous labeling, inconsistent education, and inconvenient workflow placement of receptacles, reliably lead to misclassification into RMW streams. Taken together, the literature suggests that adopting targeted waste‑recycling programs, integrating low‑flow anesthesia, and implementing sustainability‑conscious procurement policies yield greater overall reductions in environmental footprint and cost than focusing on anesthetic vapors in isolation [5-7,14,19].

However, the findings of this review must be considered in the context of several key limitations. The majority of available evidence is derived from observational audits, single-center quality improvement projects, surveys, and implementation case studies, all of which exhibit heterogeneous baselines, outcome measures, and follow-up durations. This heterogeneity limits direct comparability across institutions and may restrict generalizability to similar practice environments. Additionally, publication bias may be present, as positive or successful sustainability initiatives are more likely to be reported than neutral or unsuccessful ones, and economic outcomes are frequently presented without standardized denominators or adjustments for local cost structures. Consequently, this review offers a pragmatic framework and a range of plausible effects rather than precise pooled estimates. Future research should prioritize multicenter studies with standardized measures, extended follow-up periods, and explicit evaluation of intervention effectiveness across diverse resource settings [3,23,30].

## Conclusions

Clinicians can rapidly accelerate sustainability gains in anesthesia by combining concrete waste‑management steps with targeted emissions mitigation. Across studies and implementation reports, the most enduring obstacles are workflow barriers rather than a lack of provider intent, so interventions that give precedence to convenience and clarity, such as placing recycling receptacles at the anesthesia workstation, using simple pictogram‑based signage aligned with local recycling rules, and embedding brief recurring education into routine departmental activities, consistently increase waste diversion and lower RMW costs keeping clinical efficiency. Evidence from multi‑site pilots and single‑center quality‑improvement projects also suggests that low‑flow anesthesia protocols, preferential use of lower‑GWP agents, and, where feasible, gas‑capture technologies can be integrated with minimal interruption when supported by leadership and clear local guidelines.

Based on this review, departments seeking to improve environmental performance should (1) map local recyclability and RMW policies with input from facilities and waste vendors; (2) deploy point‑of‑use recycling at anesthesia workstations with clear pictograms and color-coded containers; (3) integrate sustainability topics into orientation, in‑service teaching, and “green rounds”; and (4) track a small set of core metrics, such as RMW volume per case, diversion rates for key materials like blue wrap and IV containers, and usage of high‑GWP agents. These steps deliver effective starting points that can be adapted to local constraints and resources rather than relying on a single “one‑size‑fits‑all” intervention.

At the same time, the evidence base has important limitations. Most available studies are observational audits, surveys, and quality improvement (QI) reports with heterogeneous baselines, outcome measures, and follow‑up periods, and economic data are often reported without standardized denominators or adjustment for local cost structures. Further studies should prioritize multicenter studies with standardized definitions and metrics, evaluate the long‑term durability of both waste‑ and gas‑mitigation interventions, and explicitly compare their environmental and financial impacts across diverse resource settings. Coupling solid‑waste recycling with gas‑emission mitigation is still a pragmatic, high‑yield pathway for clinicians to reduce the sustainability footprint and operating costs of anesthesia while building the infrastructure and culture needed for deeper decarbonization.
